# Use of Genetically Altered Stem Cells for the Treatment of Huntington’s Disease

**DOI:** 10.3390/brainsci4010202

**Published:** 2014-03-24

**Authors:** Andrew T. Crane, Julien Rossignol, Gary L. Dunbar

**Affiliations:** 1 Field Neurosciences Institute Laboratory for Restorative Neurology, Brain Research and Integrative Neuroscience Center, Program in Neuroscience, Central Michigan University, Mount Pleasant, MI 48859, USA; E-Mails: crane1a@cmich.edu (A.T.C.); rossi1j@cmich.edu (J.R.); 2 College of Medicine, Central Michigan University, Mount Pleasant, MI 48859, USA; 3 Field Neurosciences Institute, Saginaw, MI 48604, USA

**Keywords:** Huntington’s disease, mesenchymal stem cells, induced pluripotent stem cells, brain derived neurotrophic factor, cell therapy, genetic engineering

## Abstract

Transplantation of stem cells for the treatment of Huntington’s disease (HD) garnered much attention prior to the turn of the century. Several studies using mesenchymal stem cells (MSCs) have indicated that these cells have enormous therapeutic potential in HD and other disorders. Advantages of using MSCs for cell therapies include their ease of isolation, rapid propagation in culture, and favorable immunomodulatory profiles. However, the lack of consistent neuronal differentiation of transplanted MSCs has limited their therapeutic efficacy to slowing the progression of HD-like symptoms in animal models of HD. The use of MSCs which have been genetically altered to overexpress brain derived neurotrophic factor to enhance support of surviving cells in a rodent model of HD provides proof-of-principle that these cells may provide such prophylactic benefits. New techniques that may prove useful for cell replacement therapies in HD include the use of genetically altering fate-restricted cells to produce induced pluripotent stem cells (iPSCs). These iPSCs appear to have certain advantages over the use of embryonic stem cells, including being readily available, easy to obtain, less evidence of tumor formation, and a reduced immune response following their transplantation. Recently, transplants of iPSCs have shown to differentiate into region-specific neurons in an animal model of HD. The overall successes of using genetically altered stem cells for reducing neuropathological and behavioral deficits in rodent models of HD suggest that these approaches have considerable potential for clinical use. However, the choice of what type of genetically altered stem cell to use for transplantation is dependent on the stage of HD and whether the end-goal is preserving endogenous neurons in early-stage HD, or replacing the lost neurons in late-stage HD. This review will discuss the current state of stem cell technology for treating the different stages of HD and possible future directions for stem-cell therapy in HD.

## 1. Introduction

Huntington’s disease (HD) is a genetically inherited neurodegenerative disorder caused by a mutation to the huntingtin (*HTT*) gene. The mutation, an expanded CAG repeat on exon 1 of the *HTT* gene located on chromosome 4, has been thoroughly researched since its description in 1993 [1], however the cause for the disease-related pathology is not understood. Various hypotheses have emerged: loss of trophic support, glutamate excitotoxicity, mitochondrial dysfunction, and autophagy. Each of these hypotheses is valid with respect to the function of mutant *HTT* (m*HTT*), yet no single hypothesis unifies the distinct pathology of HD.

Many of the hallmark neuropathological signs of HD include a loss of GABAergic medium spiny neurons (MSNs) in the caudate nucleus and putamen. The progressive nature of HD and variability in length of the CAG repeat makes each individual case different. Adult-onset HD is typically diagnosed following the initial emergence of self-reported symptoms, including cognitive/emotional disturbances and difficulties sleeping. As the disease progresses, symptoms include ballistic or chorieform movements in the extremities, dementia, and impaired cognitive abilities. Patients in the advanced stage of the disease become unable to care for themselves and eventually die at approximately 20 years following the onset of symptoms, usually as a result of aspiration from pneumonia (for review see [2]). Various neuroimaging and post-mortem tissue analyses have identified multiple grades of neuronal loss in the HD brain, beginning with striatal atrophy and white matter loss up to 15 years prior to the onset of symptoms [3,4] followed by a 50% loss of MSNs in the caudate in the early symptomatic stages, and a 95% loss of MSNs in end-stage HD patients [5,6].

Currently, no treatment to delay or stop the progression of the disease is available. In 2008, the USDA approved tetrabenazine to treat the motor symptoms of HD, while anti-psychotics and anti-depressants have been prescribed to treat other movement as well as cognitive and emotional disturbances [7,8]. However, these treatments are only palliative and not always effective. Therefore, it is necessary to find treatments that can alleviate the symptoms and delay the progression of the disease. Investigation of stem cell therapies in recent years have examined the ability of these cells to delay the onset or progression of HD and potentially replace lost cells, yet new methods have enhanced the abilities and benefits of stem cells. This review will explore new techniques in modification of stem cells, including alterations that increase trophic factor release in mesenchymal stem cells (MSCs), MSC-mediated gene therapies, and induced pluripotent stem cells for the treatment of HD.

## 2. Increased Trophic Support

It has been well established that reductions in pro-cell survival neurotrophic factors (NTFs), such as brain derived neurotrophic factor (BDNF), is dramatically reduced in the HD brain [9,10]. The BDNF protein, produced in the cerebral cortex and transported via corticostriatal tracts to MSNs [11], is necessary for MSN survival [12].

To further understand the significance of BDNF in the striatum of the HD brain, transgenic HD mice were crossed with heterozygote BDNF knockout mice, which accelerated the progression of the disease phenotype [11,13]. Conversely, crossing HD transgenic mice with mice that overexpress the BDNF gene delayed the progression of the disease and protected against neuropathological dysfunction [14,15]. Although, it should be noted that when the HD-BDNF transgenic cross reached 16 months, the animals began to show epileptic-like seizure activity, most likely because the level of BDNF was not tolerated [15]. Delivery of therapeutically significant amounts of NTFs is challenging. Although repeated peripheral administrations of fibroblast growth factor-2 into mouse models of HD have shown some efficacy [16], delivery of many other NTFs, including BDNF, into the HD brain remains a challenge, due to the fact that many NTFs are large, polarized proteins that do not readily cross the blood-brain-barrier [17], relegating the delivery of NTFs directly into the central nervous system.

### 2.1. Overexpression in Endogenous Cells

With the knowledge that BDNF is neuroprotective, researchers have attempted to increase BDNF production from endogenous cells within the striatum via adenoviral injections [18] or through an adeno-associated virus (AAV) vector injections into the striatum [19,20]. Increases in the numbers of cells expressing dopamine- and cAMP-regulated phosphoprotein of 32 kDa (DARPP32; specific to MSNs) were observed in animals injected with an AAV-BDNF, following quinolinic acid (QA) lesions, relative to lesioned AAV-control animals [19]. Similarly, Goldman and colleagues have developed an AAV which overexpress BDNF and noggin, the latter to inhibit glial differentiation, which has shown to increase recruitment of MSNs to the striatum [20]. Injection of these AAVs has been shown to reduce motoric deficits and prolong lifespan in the transgenic R6/2 mouse model of HD [21,22,23]. Interestingly, an increase of parvalbumin expressing interneurons was observed following an injection of an AAV vector, which also increased the release of glial derived neurotrophic factor (GDNF) [19]. These findings suggest that multiple NTFs are more efficacious in protecting multiple cell types.

### 2.2. Mesenchymal Stem Cells

Originally documented by Friedenstein and colleagues [24], MSCs are multipotent stromal cells, which have since been defined by three main characteristics: (1) plastic adherence; (2) ability to differentiate into a diverse set of tissue within the mesoderm lineage; and (3) self-renewal [25]. MSCs can be derived from various sources (including bone marrow, umbilical cord blood, adipose tissue, *etc.*), and can be easily isolated and expanded *in vitro*.

**Figure 1:**
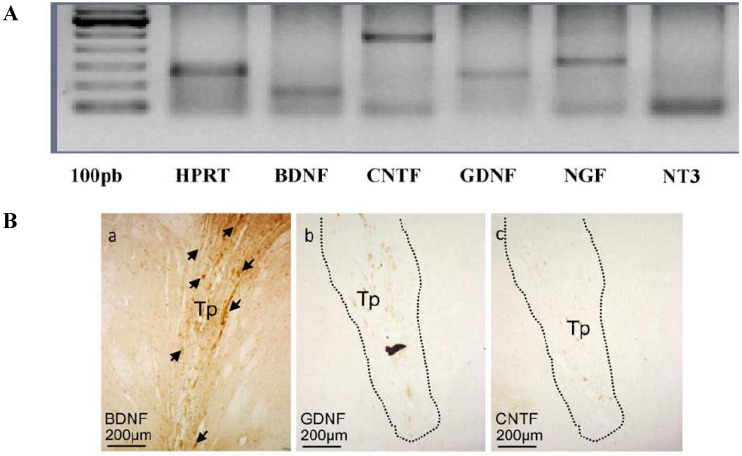
Trophic support from MSCs. (**A**) RT-PCR analysis of rat MSCs at passage 4 showing that, *in vitro*, MSCs express mRNA for BDNF (brain derived neurotrophic factor), CNTF (ciliary neurotrophic factor), GDNF (glial cell-line derived neurotrophic factor), NGF (nerve growth factor) and NT3 (neurotrophin 3). Control: HPRT; (**B**) Labeling of brain sections for BDNF (**a**, some MSCs pointed by the arrows) but not GDNF (**b**) or CNTF (**c**) show trophic factors synthesized by MSCs 72 days post-transplantation [28].

Transplantation of bone-marrow derived MSCs into rodent models of HD has been shown to reduce motor and cognitive deficits [26,27,28,29]. In 2003, Lescaudron and colleagues transplanted autologous, whole bone marrow into the brain of rats that were treated with QA [26]. Reductions in working memory errors were reported, however no neuronal differentiation of transplanted cells was observed, suggesting that beneficial effects were the result of a mechanism other than cell replacement. It was later observed that MSCs transplanted into a 3-nitropropionic acid (3-NP) rat model of HD improved latency to fall on the rotarod as well as reduced the size of the lesion to the striatum [28], which was hypothesized to be a result of NTF release from the cells (Figure 1).

The intrinsic characteristics of MSCs, such as immunomodulation and NTF release, have made these cells a target for cell-based therapies in HD. The ability of MSCs to modulate the local immune environment comes from cell-to-cell contact and through the release of interleukins and cytokines that interact with a wide range of immune cells that, in turn, help to protect transplanted MSCs from acute and prolonged immune responses [30,31,32,33]. *In vitro* characterizations of MSCs have revealed the presence of many NTFs, including BDNF, GDNF, and nerve growth factor (NGF) [28,34]. Recent advances in stem cell characterization techniques have allowed researchers to explore the secretome of MSCs, with the aim of identifying all secreted molecules which, in turn, may provide insight into the mechanisms of MSC benefits (for review [35]).

The direct correlation between intrastriatal levels of BDNF led one group to induce MSCs to overexpress BDNF and GDNF through changes in culture conditions of the MSCs *in vitro*. When transplanted prior to QA injection, these NTF cells were able to reduce lesion size, relative to non-transplanted controls [36]. Similarly, R6/2 mice transplanted with human MSCs induced to secrete NTFs displayed transient reductions in rotarod deficits, which were dependent upon age at which R6/2 mice were transplanted [37].

### 2.3. Genetic Engineering of Transplantable Cells

To facilitate a greater release of NTFs for an extended period, transplantable cells have been engineered to secrete greater than physiological levels of NTFs. In 2000, a group tested the ability of rat fibroblasts, individually engineered to overexpress a variety of NTFs, including BDNF, NT-3, or NT4/5, to protect against QA lesion [38]. The engineered cells were first transplanted into the rat striatum, followed by a QA injection 24 h after transplantation. It was observed that all rats transplanted with engineered cells reduced TUNEL labeling when observed 7 days after inducing a lesion. Furthermore, all engineered cell types differentially protected against the death of neurons expressing glutamate- decarboxylase-, preproenkephalin-, and preprotachykinin-, but not dynorphin-expressing cells. As a whole, it was observed that transplants of fibroblasts overexpressing BDNF were the most efficient in protecting against neuronal death.

Similarly, a second group of researchers transplanted astrocytes which overexpressed BDNF [39]. To this end, a transgenic mouse was created to overexpress BDNF via control of the glial fibrillary acidic protein promoter [39]. Astrocytes were isolated and expanded from the transgenic animals, then transplanted into wild-type mice, followed by QA injections at 30 days post-transplantation. Similar to previous results, the transplanted BDNF-expressing astrocytes gave protection against the toxic lesion, increasing the total number of DARPP32 and parvalbumin expressing neurons, relative to non-engineered astrocytes. Animals transplanted with BDNF-expressing astrocytes also showed a reduction in apomorphine-induced rotations.

**Figure 2:**
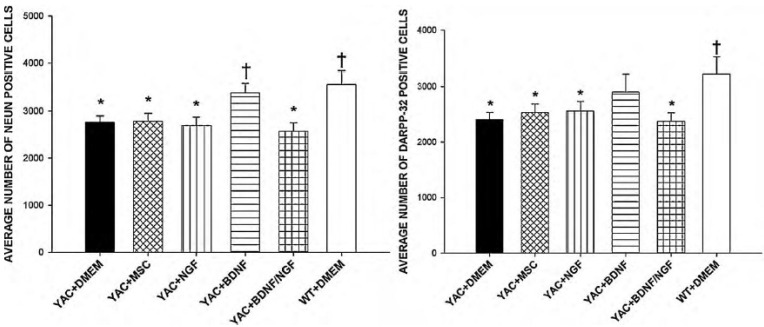
Neuroprotective effects of NTF secreting MSCs. Sparing of NeuN-positive (left) and Darpp-32-positive (right) cells was observed in YAC 128 mice that received striatal transplants of mesenchymal stem cells (MSCs) that were genetically altered to over-express brain derived neurotrophic factor (YAC + BDNF), but not in mice that received transplants of MSCs that were genetically modified to over-express nerve growth factor (YAC + NGF), a combination of MSCs that over-expressed both NGF and BDNF (YAC + BDNF/NGF), or unaltered MSCs (YAC + MSC). Note: * indicates a significant difference from WT + DMEM; ^†^ indicates a significant difference from YAC + DMEM [40].

Both fibroblasts and astrocytes, that were engineered to overexpress BDNF, were efficient in protecting against QA induced HD-like pathology, but were not tested in a transgenic animal model of HD. Given that MSCs are easy to isolate and expand, our lab utilized bone-marrow derived MSCs, and then retrovirally transfected to overexpress BDNF, NGF, or a combination of BDNF and NGF [40]. These cells were then transplanted into the YAC128 transgenic mouse model of HD, and observed for 9 months. All YAC128 mice that were transplanted with the engineered MSCs had reductions in disease pathology, with an increased latency to fall from the rotarod and reduced clasping scores. Compared to all engineered cell lines, BDNF-expressing MSCs showed the highest amount of behavioral protection. Neuropathologically, YAC128 mice transplanted with BDNF-expressing MSCs displayed a normalization of total neurons within the striatum, and protected against significant losses of DARPP32 neurons (Figure 2).

These transplantation studies, along with the results viral-vector-mediated increases in BDNF levels have shown that treatments of this nature are protecting endogenous neurons against cell death. With the promising results from the BDNF expressing MSCs [40] and lentiviral safety [41], the California Institute for Regenerative Medicine (CIRM) granted the University of California at Davis to take lentivirally engineered MSCs to overexpress BDNF to the clinic, with the goal of creating an FDA-approved cellular therapy for HD.

It is generally accepted that MSCs do not differentiate into neurons following transplantation. Thus, the use of stem-cell mediated transfer of NTFs into the striatum of HD patients will provide the most benefit early in disease progression, when high numbers of MSNs are still present in the striatum.

## 3. Gene Therapy

Gene therapy is a promising new avenue for the treatment of HD. Due to the relative simplicity of a single mutation on a single gene, multiple strategies to reduce the amount of the mutated protein have evolved in recent years. However, the basic properties of the HTT protein and the ubiquitous expression of HTT in all cells has provided challenges to gene therapies. A major obstacle is that HTT is necessary for normal development. This has been shown by Duyao and colleagues who found that *Htt* null mice die at embryonic day 7.5 [42]. Furthermore, conditionally knocking out wild-type *Htt* in adult mice causes progressive degeneration and sterility [43], demonstrating that potential therapies must aim at reducing mHTT expression.

One strategy to reduce levels of the mutated protein is the use of proteins that work intracellularly to bind to target proteins, or intrabodies. When the intrabodies bind to mHTT, the complex is ubiquitinated, marking it for degradation and removal. Two particular intrabodies, Happ1 and EM48, introduced into the striatum via viral vectors are able to reduce some of the neuropathological and behavioral symptoms in HD mice [44,45,46,47]. However, the ability to effectively deliver intrabodies, specificity to mHTT, and to prevent cytoplasmic protein misfolding, as well as to promote long-term symptom reduction are still unclear and require further investigation [48].

### 3.1. RNA Interference

A second strategy is to reduce expression of m*HTT* via silencing expression through RNA interference (RNAi). This technology employs the endogenous RNA-induced silencing complex, which binds the anti-sense micro-RNA, or interfering RNA, to mRNA which then either cleaves or destroys the double stranded RNA. The most commonly employed RNAi methods have been virally introduced into the brain through AAV vectors [49,50,51,52]. In healthy non-human primates AAVs delivering microRNA against wild-type *HTT* in the rhesus macaque showed a 45% reduction in *HTT* within the putamen, with no adverse events noted [52].

In mouse models of HD, studies showed a 50% reduction in *Htt* expression over 5 months after injection of the virus into the N171-82Q knock-in model of HD [49] and upwards of 80% reduction of *HTT* in the R6/1 mouse model of HD [50]. In an effort in improve integration, DiFiglia and colleagues conjugated a short interfering RNA (siRNA) targeting *HTT* with cholesterol, which produced a trend towards increasing cellular uptake [51]. This cholesterol-conjugated siRNA reduced clasping scores and footslips in an AAV mouse model of HD, as well as a reduction in aggregation of mHTT at two weeks post-infection. When cholesterol-conjugated siRNA was injected into neonatal R6/2 mice, a robust delay in behavioral deficits was observed [53]. Taken together, RNAi has shown promise when virally infecting endogenous cells.

### 3.2. Genetic Engineering of MSCs

A major downfall of the RNAi technology, however, is the relatively short half-life of RNA: Coupled with low infection rates of viral vectors and the toxicity of unregulated RNAi, new methods of continuous delivery of RNAi to target tissue still needs to be refined. As previously noted, MSCs possess beneficial properties when transplanted in the HD brain, providing a micro-environment suitable for supporting the degenerating brain, as well as long-term survival post-transplantation. It has been established that MSCs can rescue cellular dysfunction caused by mitochondrial depletion by the transfer of mitochondria through cytoplasmic extensions [54], and that RNA and other nucleic acids can be transferred by various cell-to-cell mechanisms [55,56,57]. Olson and colleagues tested the ability for MSCs transfected with a short hairpin RNA (shRNA) against *HTT* on reducing expression *in vitro* [58]. After transfection, MSCs were characterized through flow cytometry, growth curve analysis, differentiation capabilities, and karyotype analysis. They found that all assays confirmed that the MSCs that were engineered to express shRNA were karyotypically normal, with no adverse effects on cell proliferation, nor was the ability to differentiate into bone, cartilage, or adipose tissue compromised. When co-cultured with cells expressing m*HTT*, the engineered MSCs were able to reduce expression of mutated mRNA and protein. This suggests that these effects were mediated through either cell-to-cell contact or other mechanisms, and that MSCs have the capability of reducing m*HTT* in the brain.

## 4. Pluripotent Stem Cells

Pluripotent stem cells have garnered much attention in neurodegenerative disorders for their potential to replace lost neurons. Pluripotent cells are derived from the inner cell mass of a blastocyst. These cells express the transcription factors OCT4 [59] and SOX2 [60]. Relative to MSCs or neural stem cells (NSCs), which are lineage restricted, pluripotent cells are able to form any tissue in the three germ lineages (ectoderm, endoderm, and mesoderm).

Obtaining true pluripotent stem cells suitable for therapeutic transplantation has raised concerns, both ethically and practically, because discarded embryos must be donated after *in vitro* fertilization and must be expanded in order to obtain the quantity of cells suitable for transplantation.

### 4.1. Fetal Transplantation

Cells derived from the ganglionic eminence have been utilized with the hope that the fetal cells destined to become cells of the basal ganglia will differentiate into MSNs. Long-term clinical studies, where cells from the ganglionic eminence were transplanted into HD patients have shed light on the variability associated with fetal transplants. Bachoud-Levi and colleagues transplanted ganglionic eminence tissue into the striata of HD patients and found that 3 of the 5 patients displayed a delay in the progression of the disease and showed metabolically active tissue at 10 years following transplantation [61,62,63].

However, these beneficial effects were not seen in all patients receiving transplantations of fetal ganglionic eminence. Other groups observed short-term graft survival as well as HD-related pathology, such as neurodegeneration and aggregate formation within the graft [64,65]. These results offer insight into clinical applications of fetal grafts, but the logistics of transplantation into HD patients necessitate the need for cells that are more suitable for transplantation.

### 4.2. Induced Pluripotent Stem Cells

In 2006, Yamanaka and colleagues published a seminal paper showing that somatic cells could be reprogrammed by retroviral transfection of embryonic transcription factors OCT3/4 and SOX2, as well as embryonic stem cell (ESC) maintenance transcription factors KLF4 and c-MYC [66] (commonly termed the “Yamanaka factors”). These induced pluripotent stem cells (iPSCs) exhibited much of the same characteristics of embryonic cells, including morphology and genes. Furthermore, when transplanted into nude mice, these iPSCs formed teratomas of all three germ lineages. Since 2006, much research has been put into iPSC development, primarily towards generating new methods of inducing pluripotency and understanding HD.

### 4.3. Disease Modeling

In the disease modeling field, the ability to take somatic cells carrying the genetic mutation for HD and to reprogram these cells into iPSCs with the Yamanaka factors has allowed researchers to further elucidate the mechanisms of HD, as well as create novel therapeutics aimed at reducing the cellular pathology of HD [67,68] or correcting the genetic mutation [69,70]. The HD iPSC Consortium is a multi-institution, multi-national collaborative effort that has generated and characterized 14 different iPSC lines from HD patients with a variety of different CAG repeat lengths [68]. Through cellular and molecular assays, this group identified a variety genes that are differentially expressed in the HD iPSCs, as well as CAG-repeat-dependent phenotypes that were not present in control iPSC lines. However, much of the observed effects were observed in iPSC lines in which the CAG repeats are observed only in the less commonly occurring juvenile form of HD (100 + CAGs).

### 4.4. Transplantation of iPSCs

Transplantation of iPSCs into the central nervous system for therapeutic use is still an emerging topic of interest. Many practical hurdles, such as the propensity of iPSCs to form tumors within the transplant site when permanently integrated oncogenes (*KLF4* and *c-MYC*) are used, as well as the potential for transplanted iPSCs develop the HD phenotype (as seen by fetal graft transplants [65]), or rejection of iPSCs by the immune system.

In 2012, Jeon and colleagues generated iPSCs from a juvenile form of HD (72 CAG repeats) and tested the potential of these cells to differentiate into MSNs *in vitro* and *in vivo* [71]. Following a standard *in vitro* multi-stage differentiation protocol, iPSCs were able to form neural progenitors that expressed nestin, as well as mature neuronal cells that expressed NeuN, GABA, and DARPP32. Patch-clamp analysis confirmed MSN phenotype, through GABA-induced whole cell current profiles. One week following intrastriatal QA administration to rats, iPSCs that had been differentiated into the neural progenitor state, were transplanted into the affected straita. Behavioral recovery was observed as measured by reductions in apomorphine-induced rotations and in the ability to grasp food pellets in the staircase test, beginning 4–6 following transplantation. Histological analysis revealed that transplanted cells expressed a variety of neuronal lineage proteins, including nestin, MAP-2, DARPP32, and GABA, at 12 weeks following transplantation. Importantly, these HD-derived iPSCs did not show aggregate formation. The authors were not able to rule out the possibility that the benefits they observed were due to non-cell autonomous effects (such as trophic support), but these results are promising, by indicating iPSCs may provide an effective alternative to ESCs and fetal transplants.

**Figure 3:**
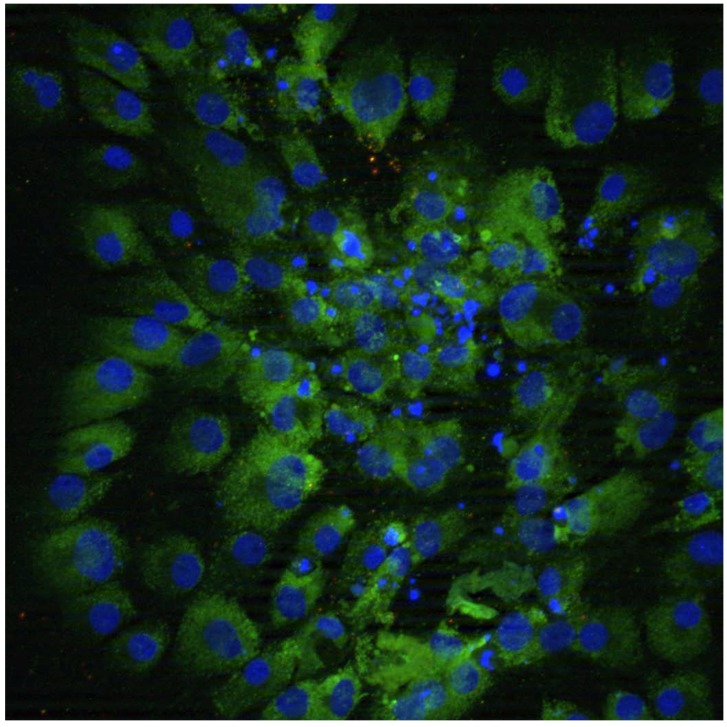
Immunocytochemical analysis of iPSCs *in vitro.* Immunocytochemistry revealed adenovirus-generated iPSCs (blue) can be differentiated into neural rosettes *in vitro*, expressing NCAM (green).

Our lab has focused on the development of therapeutically relevant iPSCs for the treatment of HD. To this end, a pair of non-integrative adenoviruses expressing the Yamanaka factors (*Oct4/Klf4/Sox2* and c-*Myc*) were inserted into rat tail tip fibroblasts [72]. This novel method generated iPSCs with equal efficiency as established lentiviral methods as well as the ablity to differentiate into neural rosettes *in vitro* (Figure 3).

**Figure 4:**
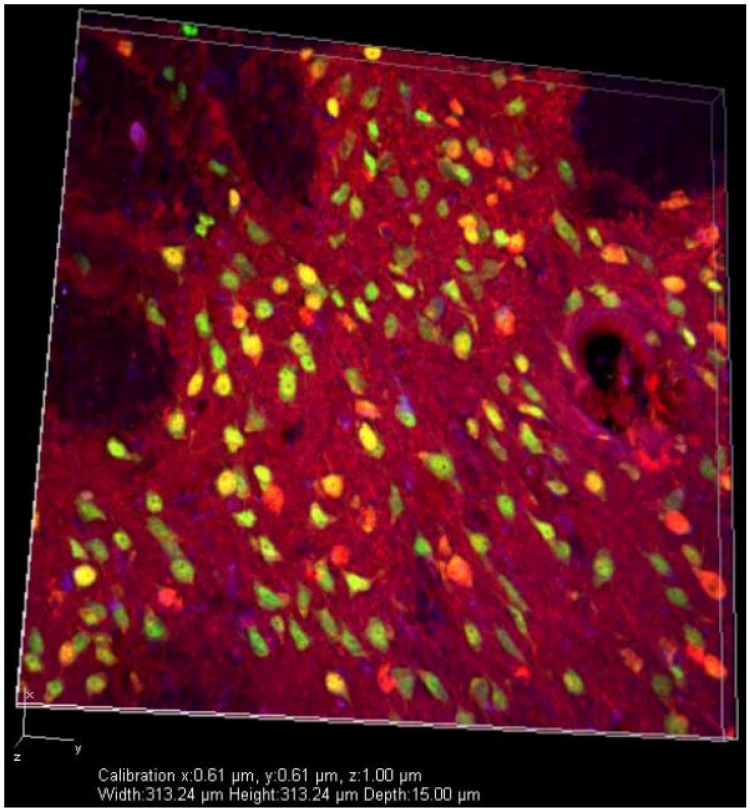
Confocal image of iPSCs transplanted in the 3-NP rat brain: Immunohistochemical analysis of transplanted iPSCs (blue), mature neurons (NeuN; green), and medium spiny neurons (DARPP32; red). Transplanted rats displayed surviving iPSCs (blue) around the injection site at the conclusion of the study.

In the undifferentiated state, iPSCs were then transplanted into immune-competent rats. Transplanted cells were observed up to 90 days post-transplantation, with some of the iPSCs expressing markers of mature, region-specific, neuronal phenotypes. Importantly, the formation of tumors was not observed. Given these promising results, adenovirus-generated iPSCs were transplanted into the striata of rats given progressive doses of 3-NP which replicates the emergence of deficits that are analogous to HD [73]. In the 3-NP rats that received transplants at early- and mid-stage of disease phenotype, behavioral sparing of motor performance on the accelerod was observed. Surprisingly, a recovery of function was observed when 3-NP rats were transplanted at end-stage. Histological analysis of brains following transplantation showed survival of iPSCs with mature neuronal phenotypes (Figure 4), as well as a reduction in neuronal loss due to 3-NP administration. To further elucidate the mechanisms of action in transplanted rats, we observed that mRNA levels of BDNF and tumor necrosis factor alpha were not differentially regulated, suggesting the observed effects were not due to BDNF-induced neuroprotection or immune regulation by tumor necrosis factor-alpha.

Results from these studies, while still preliminary, address some of the concerns regarding biosafety of iPSCs and transplanting these cells into the HD brain, which showed no signs of graft rejection or overgrowth of transplanted cells at the site of transplantation. Unlike therapies aimed at increasing BDNF or reducing the expression of m*HTT*, where earlier interventions will be more beneficial in delaying the progression of the disease, iPSC therapy has the potential to reduce HD symptoms at mid- and late-stages through the replacement of lost neurons.

## 5. Future Directions

Much of what has been presented here is pre-clinical research focusing on new technologies aimed at alleviating the major symptoms observed in HD and extending the shortened lifespan. As HD is a multi-faceted disease, affecting more than just the brain, therapies that treat the total effects of the disease may prove to be necessary. However, as of now, no such therapy exists. Given that the currently tested therapies aim at single solution, combinatory treatments may prove to be more efficacious.

Our lab has utilized the immunomodulatory and NTF support of MSCs and co-transplanted them with NSCs, which possess the ability to differentiate into neurons, into the transgenic rat model of HD [74]. In this paradigm, it was observed that HD rats receiving transplants of both MSCs and NSCs had a greater behavioral sparing effect on the rotarod. It was also reported that transplants of MSCs increased the surviving graft size of NSCs, suggesting that the microenvironment provided by the MSCs allowed for a greater survival of NSCs.

In order to maximize therapeutic potential of transplantable cells, several groups have worked toward pushing stem cells further en route to becoming MSN progenitor cells prior to transplantation [75,76,77]. Through chemically defined media, ESCs, NSCs, and iPSCs have been engaged to differentiate into DARPP32 expressing cells. As one might expect, an important factor involved in the differentiation of MSNs is the presence of BDNF [69,70].

Given these data, a logical next step would be a co-transplantation paradigm using iPSCs engaged towards a MSN fate with MSCs engineered to overexpress BDNF. It would be hypothesized that the immunomodulatory cytokines released by the MSCs would enhance graft survival. Further, the BDNF released by the genetically engineered MSCs would enhance differentiation of the iPSCs into MSNs.

## 6. Conclusions

The past decade has provided many new insights and methods in molecular biology, allowing researchers to adapt techniques which are best suited for a particular cause. As HD is a progressive neurodegenerative disorder, therapies aimed at treating patients at different stages of the disease are being pursued. Early- and mid-stage HD patients already see a marked loss of MSNs within the caudate nucleus and putamen as well as dramatic decrease in BDNF, while late-stage HD patients have lost an overwhelming number of neurons within the basal ganglia and cerebral cortex. Early interventions with MSCs engineered to overexpress the trophic factor BDNF or RNAi show the ability to protect against neurodegeneration, while later interventions with iPSCs are aimed at cell-replacement. Whether these therapies will advance towards the clinic individually, or if a combination of treatments will prove more effective, remains to be seen. However, the rate at which new advances in technology are occurring will certainly provide numerous therapeutic options in the near future.

## Abbreviations

3-NP: 3-nitropropionic acid
AAV: adeno-associated virus
BDNF: brain derived neurotrophic factor
DARPP32: Dopamine and cAMP regulated phosphoprotein of 32 kDa
GDNF: glial derived neurotrophic factor
ESC: embryonic stem cell
HD: Huntington’s disease
*HTT*/HTT: huntingtin gene/protein
iPSC: induced pluripotent stem cell
m*HTT*: mutant huntingtin
MSC: mesenchymal stem cell
MSN: medium spiny neuron
NSC: neural stem cell
NTF: neurotrophic factor
NGF: nerve growth factor
RNAi: RNA interference
siRNA: short-interfering RNA
shRNA: short-hairpin RNA
QA: quinolinic acid
